# Analyses of the Survival Time and the Influencing Factors of Chinese Patients with Prion Diseases Based on the Surveillance Data from 2008–2011

**DOI:** 10.1371/journal.pone.0062553

**Published:** 2013-05-06

**Authors:** Cao Chen, Ji-Chun Wang, Qi Shi, Wei Zhou, Xiao-Mei Zhang, Jin Zhang, Chan Tian, Chen Gao, Xiao-Ping Dong

**Affiliations:** 1 State Key Laboratory for Infectious Disease Prevention and Control, National Institute for Viral Disease Control and Prevention, Chinese Center for Disease Control and Prevention, Beijing, People’s Republic of China; 2 Division of Science and Technology, Chinese Center for Disease Control and Prevention, Beijing, People’s Republic of China; 3 Chinese Academy of Sciences, Key Laboratory of Pathogenic Microbiology and Immunology, Institute of Microbiology, Chinese Academy of Sciences, Beijing, People’s Republic of China; Deutsches Zentrum für Neurodegenerative Erkrankungen e.V., Germany

## Abstract

**Background:**

Prion diseases are kinds of progressive, incurable neurodegenerative disorders. So far, survival time of the patients with these diseases in China is unclear.

**Methods:**

Based upon the surveillance data from Chinese Creutzfeldt-Jakob disease (CJD) surveillance network from January 2008 to December 2011, a retrospective follow-up survey was performed. The survival times of Chinese patients with prion diseases and the possible influencing factors were analyzed.

**Results:**

Median survival time of 121 deceased patients was 7.1 months, while those for sporadic CJD (sCJD), familial CJD (fCJD) and fatal familial insomnia (FFI) cases were 6.1, 3.1 and 8.2 months, respectively. 74.0% of sCJD patients, 100% of fCJD cases and 91.7% FFI cases died within one year. The general socio-demographic factors, abnormalities in clinical examinations, clinical manifestations, and social factors did not significantly influence the survival times of Chinese prion patients.

**Conclusions:**

Survival time of Chinese patients with prion diseases was comparable with that of many Western countries, but obviously shorter than that of Japan. Patients with acute onset and rapid progression had significantly short survival times.

## Introduction

Transmissible spongiform encephalopathies (TSEs), or prion diseases, are a group of neurodegenerative disorders that afflict human and animals [1]. Human prion diseases consist of three primary types, including sporadic, genetic and acquired. Sporadic human prion disease mainly indicates sporadic Creutzfeldt-Jakob disease (sCJD) which is the most common condition and its reasons are still unclear. Genetic prion diseases, such as familial Creutzfeldt-Jakob disease (fCJD), Gerstmann-Sträussler-Scheinker syndrome (GSS), and fatal familial insomnia (FFI), are caused by a sort of mutations within human PrP encoding *PRNP* gene. Acquired Creutzfeldt-Jakob disease traditionally refers to the diseases transmitted by various iatrogenic procedures, such as treatment with human pituitary growth hormones, dura mater and cornea grafts, deep brain electrodes and neurosurgery [2]. Another acquired CJD, namely variant Creutzfeldt-Jakob disease (vCJD), is caused by consuming the beef or its products contaminated with the agent of bovine spongiform encephalopathy (BSE) [3].

For the great impact of the outbreak of BSE and emerging of vCJD on public health, a surveillance program for CJD has been conducted in China since 2006, which is supported by Chinese Center for Disease Control and Prevention (CCDC) [4]. Totally 811 suspected cases have been referred to CCDC, among which 219 are diagnosed as sCJD, 30 are genetic prion diseases with *PRNP* sequencing confirmation. As the pathogen of prion diseases localizes in the central nervous system (CNS), the definite diagnosis of various subtypes of human prion diseases still requires neuropathological examination and/or determination of the pathological isoform of the prion protein (PrP^Sc^) in central nerve tissues, either at brain biopsy or autopsy [5]. Probably due to the Chinese tradition, postmortem is rarely accepted in China, which leads to most sCJD cases remain at the probable diagnostic level. It reflects that a continuous follow-up survey is essential for CJD surveillance.

In this study, we presented the survival times of the patients with sCJD or genetic prion diseases from 2008 to 2011 identified by the China CJD surveillance program after a follow-up survey. Meanwhile, we investigated the relationship of the survival times with other possible influencing factors.

## Methods

### Ethics Statement

This surveillance program has been approved by Ethical Review Committee of National Institute for Viral Disease Prevention and Control, China CDC. All signed informed consents have been collected and stored by the China CJD Surveillance Center.

### Follow-up and Data Collection

**Figure 1:**
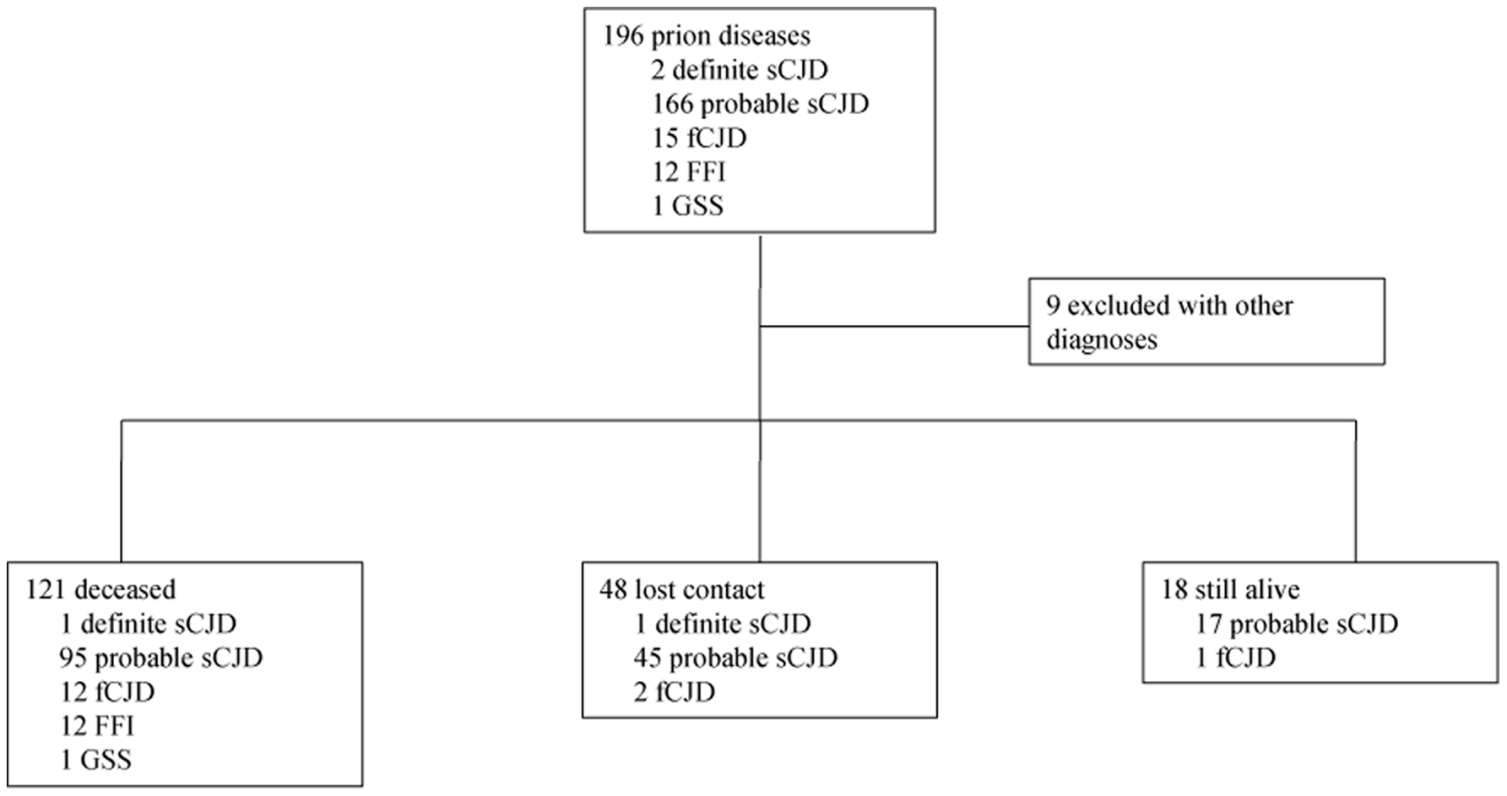
Summarization of the follow-up results of surveillance cases which initially diagnosed as various subtypes of prion disease from January 2008 to December 2011.

Suspected CJD cases referred to China CJD surveillance were diagnosed and subtyped according to the diagnostic criteria issued by CCDC, which was constituted based on the diagnostic criteria for CJD issued by WHO [6]. Totally 607 referrals from January 2008 to December 2011, among them 196 patients were diagnosed as different subtypes of prion diseases, including 2 definite sCJD, 166 probable sCJD, 15 fCJD, 12 FFI and 1 GSS. A telephone-based follow-up survey was conducted by the staff of China CJD program with the designed questionnaires. To the end of August 2012, 121 out of 196 enrolled cases already deceased, 18 cases were still alive (survival time less than 2 years: 11 probable sCJD and 1 fCJD; less than 3 years: 6 probable sCJD), 48 cases were lost to contact (1 definite sCJD, 45 probable sCJD and 2 fCJD), and 9 cases had other diagnoses (Table S1, Figure 1). Survival time was defined as the period from disease onset to death. Median survival time was calculated with the data of 121 decedents.

### Statistic Assays

Kaplan–Meier survival analysis was conducted for 121 deceased patients. Statistical analysis was conducted with SPSS version 16.0 for Windows.

## Results

### Survival Time of Patients with Various Subtypes of Prion Diseases

**Figure 2:**
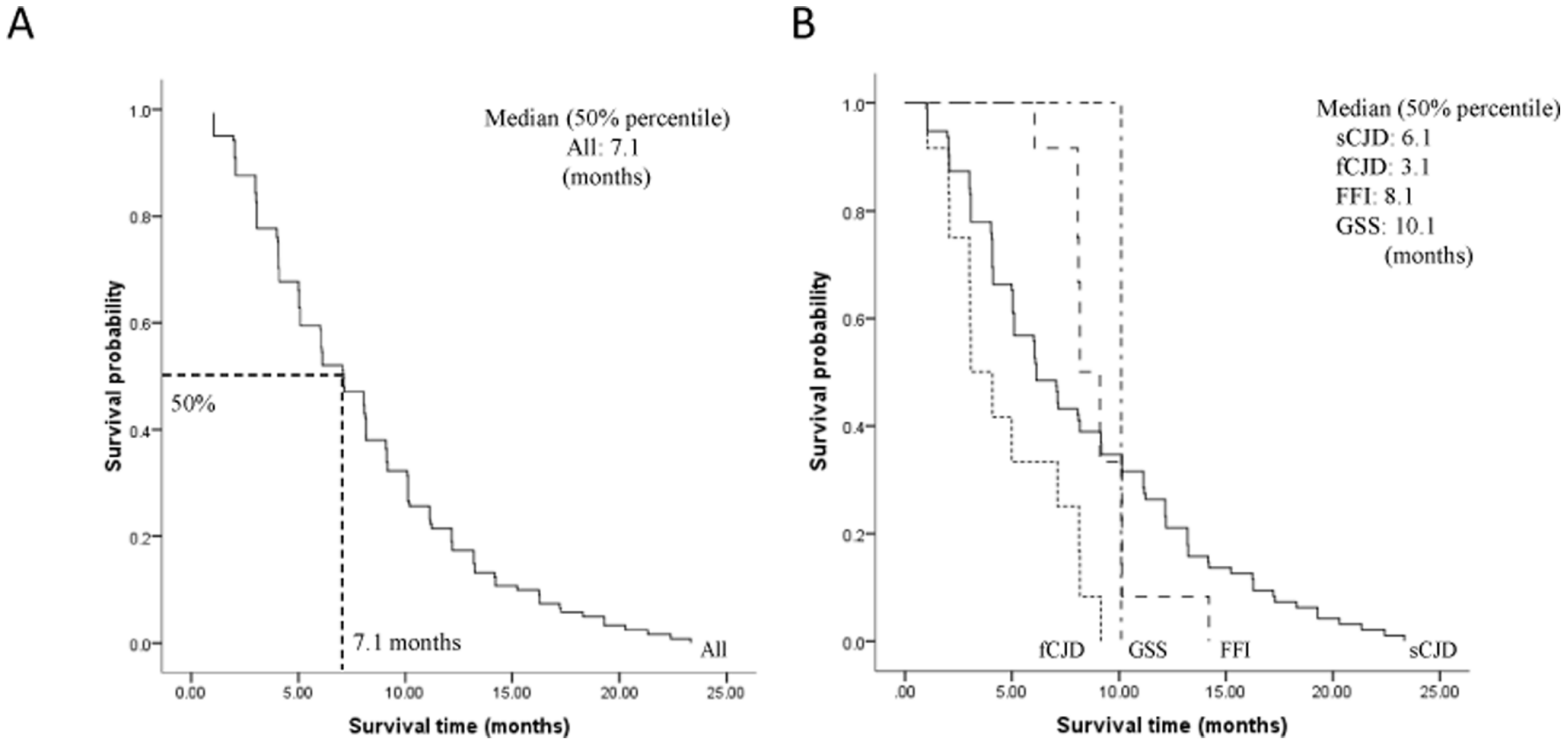
The Kaplan-Meier survival curves for deceased patients. (A) Survival time for deceased patients. B) Survival time for deceased patients stratified by subtypes of prion disease. X-axis represents survival time (months) and Y-axis represents survival probability. Graphic symbol shows the median survival time (50% percentile) of the entire and distinct subtype disease of deceased patients.

In the fatal cases (n = 121), 96 were sCJD (79.4%), 12 were fCJD (9.9%), 12 were FFI (9.9%) and one was GSS (0.8%). Kaplan-Meier survival curve for all deceased patients was shown in Figure 2A. The median survival time of all deceased cases was 7.1 months. The cumulative incidences with the survival time less than 3, 6, 12 and 24 months were 15.7%, 40.5%, 78.5% and 100%, respectively.

Kaplan-Meier survival curves for the patients stratified by subtypes of prion diseases were shown in Figure 2B. The median survival times for cases with sCJD, fCJD and FFI were 6.1, 3.1 and 8.2 months, respectively. The cumulative incidences of sCJD with the survival time less than 3, 6, 12 and 24 months were 15.6%, 42.7%, 74.0% and 100%, respectively. Since sCJD occupied the majority of the enrolled cases, the survival curve of sCJD was quite similar to that of all patients. The median survival time of fCJD patients (3.1 months) was significantly shorter than those of FFI cases (8.1 months, *P* = 0.001) and sCJD cases (6.1 months, *P* = 0.004). Particularly, all of fCJD patients died within 1 year after onset. In contrast, FFI patients seemed to have relatively long survival time. Additionally, there was only one GSS case reported who died 10.1 months later after onset.

### Relevance to General Socio-demographic Features

Out of 121 fatal cases, 65 were males and 56 were females. As shown in Table 1, median survival times of prion diseases in males and females were 7.2 (range: 1.0–23.3) and 6.1 (range: 1.0–22.4) months. Further analyses based on the subtypes of prion diseases did not reveal statistical difference between two genders. Similarly, in both genders the median survival times of fCJD patients were significantly shorter and those of FFI cases were significantly longer.

**Table 1:** Sociodemographic Features and the Median Survival Times (Months) of the Fatal Cases of Various Prion Diseases.

	All	Subtype				*P* a
		sCJD	fCJD	FFI	GSS	
Gender						
Male (*n*)	65	51	8	6	0	
Median(*range*)	7.2(1.0–23.3)	7.2(1.0–23.3)	3.0(1.0–8.2)	8.1(6.1–10.1)	–	0.005
Female (*n*)	56	45	4	6	1	
Median(*range*)	6.1(1.0–22.4)	5.1(1.0–22.4)	3.1(3.0–9.1)	9.1(8.1–14.2)	10.1	0.671
* P* b	0.444	0.216	0.342	0.363		
Age at onset						
<50 (*n*)	21	10	2	8	1	
Median(*range*)	8.2(1.0–22.4)	5.1(1.0–22.4)	4.1(4.1–9.1)	9.1(6.1–14.2)	10.1	0.829
50–70 (*n*)	77	68	5	4	0	
Median(*range*)	7.1(1.0–23.3)	7.1(1.0–23.3)	3.0(2.0–5.0)	8.1(8.1–10.1)	–	0.001
>70 (*n*)	23	18	5	0	0	
Median(*range*)	6.0(1.0–21.3)	4.1(1.0–21.3)	7.1(1.0–8.2)	–	–	0.258
* P*	0.742	0.819	0.110	0.369		

Abbreviations: sCJD = sporadic Creutzfeldt-Jakob disease; fCJD = familial CJD; GSS = Gerstmann-straussler-Sheinker syndrome; FFI = fatal familial insomnia.

a Difference in median survival time among multiple-group were tested by using Breslow (Wilcoxon) method.

b Difference in median survival time between two groups were tested by using log-rank (Mantel-Cox) method.

Median age at onset of 121 fatal cases was 61 years old (range: 19–82 y) and 82.6% of those patients were elder than 50 years. The median age at onset of FFI cases (42 years old) was obviously younger than that of sCJD cases (64 years old) and fCJD (62 years old). To explore the potential influence of onset age on the survival time, we divided the 121 patients into three groups, <50, 50–70 and >70 years old. No statistical difference in the survival time of prion diseases among various groups was addressed, though the median survival time of younger patients was relatively longer. Large portion (70.8%) of sCJD patients was in the group of 50–70 years old with relatively longer median survival time (7.1 months), but without statistical difference compared with other two groups. Among 12 fCJD cases, 8 were T188 K mutant cases with short median survivaltime (3.0 months), which may influence the survival time of the group of fCJD patients. In contrast, FFI cases usually had younger onset ages, but longer median survival times (9.1 months in the group of <50 y and 8.1 months in that of 50–70 y). Further analyses the individual information highlighted that the patients with relatively rapid onset (from the symptoms appearance to first doctor-visiting) had short survival times.

### Relevance to Ancillary Clinical Examinations and Laboratory Test

Eighty patients in the context of prion diseases undertook EEG examinations and 58 (72.5%) had periodic sharp wave complexes (PSWC). Median survival time of PSWC-negative patients was relatively longer than that of PSWC-positive patients, without significance (Table 2, *P* = 0.331). In the subtypes of sCJD and fCJD, median survival time of PSWC-negative groups was shorter than that of PSWC-positive ones (*P* = 0.223 and *P* = 0.448, respectively), whereas all FFI cases were PSWC-negative with significantly long median survival time (*P* = 0.001). Due to the distributing deviation of EEG results among the different subtypes of prion diseases, the patients with PSWC-negative in the context of prion diseases had relatively long survival time.

**Table 2:** Clinical Examinations and the Median Survival Times (Months) of the Fatal Cases of Various Prion Diseases.

	All	Subtype				*P*
		sCJD	fCJD	FFI	GSS	
PSWC^a^ on EEG^b^						
Positive (*n*)	58	54	3	0	1	
Median(*range*)	6.1(1–22.4)	6.1(1–22.4)	5.0(1.0–8.2)	–	10.1	0.410
Negative (*n*)	22	7	4	11	0	
Median(*range*)	8.1(2.1–14.2)	5.0(2.1–9.1)	3.0(2.1–7.1)	9.1(6.1–14.2)	–	0.001
*P*	0.331	0.223	0.488			
MRI^c^						
Typical (*n*)	40	36	4	0	0	
Median(*range*)	5.1(1.0–19.3)	6.1(1.0–19.3)	2.1(2.0–3.1)	–	–	<0.001
Atypical (*n*)	60	45	5	10	0	
Median(*range*)	8.1(1.0–21.3)	7.1(1.0–21.3)	7.1(3.0–9.1)	9.1(8.1–14.2)	–	0.362
*P*	0.081	0.211	0.022			
14-3-3						
Positive (*n*)	90	76	8	6	0	
Median(*range*)	6.1(1.0–23.3)	6.1(1.0–23.3)	3.0(1.0–8.2)	8.1(6.1–10.1)	–	0.002
Negative (*n*)	27	17	4	6	0	
Median(*range*)	8.1(2.0–20.3)	6.1(2.0–20.3)	7.1(4.1–9.1)	8.2(8.1–14.2)	–	0.394
*P*	0.832	0.943	0.080	0.505	–	

a Periodic sharp curve complexes.

b Electroencephalograms.

c Magnatic Resonance Imaging releases high signal in caudate/putamen.

Totally 100 deceased patients had been examined by MRI and 40 cases (40%) showed intensive signal in caudate/putamen. The median survival time of the group with typical MRI images (5.1 months) in the context of prion diseases was shorter than that of atypical MRI (8.1 months), but without significance (Table 2, *P* = 0.081). In the group of fCJD, the patients with typical MRI abnormality had significantly shorter median survival time than those of atypical image (*P* = 0.022). Similar tendency was seen in the group of sCJD, but without statistical difference (*P* = 0.221). None of FFI cases released typical MRI findings.

Out of 121 decedents, 117 performed the detection of 14-3-3 protein in CSF and 90 cases (76.9%) were positive (Table 2). In the entire patients, the median survival time of the group of 14-3-3 positive (6.1 months) slightly shorter than that of 14-3-3 negative (8.1 months), without significance (*P* = 0.832). In the subtypes of sCJD and FFI, the patients with 14-3-3 positive had comparable median survival times as those with 14-3-3 negative. In the subtype of fCJD, the patients with 14-3-3 positive seemed to have relatively shorter clinical duration, but without statistical difference compared with those with 14-3-3 negative. Compared with those of 14-3-3 positive in sCJD and FFI, the patients of 14-3-3 positive in fCJD had significantly short median survival time (*P* = 0.002).

One hundred and eleven patients undertook *PRNP* analyses, 110 cases (99.1%) were the Methionine homozygous genotype at codon 129 (M/M) and only one (0.9%) was heterozygous for Methionine/Valine (M/V) who was diagnosed as probable sCJD and died 4.1 months later after onset.

### Economic Condition and Continuous Medical Care

Currently in China, the economic situations and health insurances of the persons from urban area and countryside still varied obviously, which usually influenced largely on the treatment and medical care. Total 121 deceased patients were grouped based on their permanent residences (Urban represents relative good financial situation and Countryside represents relatively poor), as well as the disease subtypes. No statistical difference in the survival times between the patients in the groups of urban and rural area was figured out, either in the context of the entire prion diseases or in each disease subtypes (Table 3). Furthermore, the patients were divided into two groups based on the subsequent medical care after diagnosis, one was the patients who received medical care in hospital continuously (Yes represents died in hospital), and the other was those discharged from hospital soon after diagnosis and stayed at home (No represents died at home). No statistical difference in survival times was addressed between those two groups, though the median survival time of the group without continuously medical care looked slightly longer.

**Table 3:** Economical Conditions and the Median Survival Times (Months) of the Fatal Cases of Various Prion Diseases.

	All	Subtype				*P*
		sCJD	fCJD	FFI	GSS	
Permanent residence source					–	
Urban (*n*)	84	70	6	8	0	
Median (*range*)	6.1 (1.0–21.3)	6.1 (1.0–21.3)	5.0 (1.0–9.1)	8.2 (6.1–14.2)	–	0.327
Rural (*n*)	37	26	6	4	1	
Median (*range*)	8.1 (1.0–23.3)	7.2 (1.0–23.3)	3.0 (2.0–8.2)	8.1 (8.1–10.1)	10.1	0.029
* P*	0.607	0.227	0.219	0.626	–	
Continuous medical care						
Yes (*n*)	88	67	11	9	1	
Median (*range*)	6.1 (1.0–23.3)	6.1 (1.0–23.3)	4.1 (1.0–9.1)	8.2 (6.1–14.2)	10.1	0.114
No (*n*)	33	29	1	3	0	
Median (*range*)	9.1 (2.0–19.3)	10.1 (2.0–19.3)	2.0	9.1 (8.1–9.1)	–	<0.001
* P*	0.176	0.254	0.038	0.587	–	

### Relevance to Clinical Manifestations of sCJD

The clinical manifestations of various subtypes of human prion disease may vary largely. Considering limited numbers of human genetic prion diseases in the current study, we utilized only sCJD cases for further analysis in order to find possible relationship between clinical symptoms and survival time. The distribution of the foremost symptoms of 96 fatal sCJD cases was summarized in Table 4. Overall, progressive dementia was the most common foremost symptoms that were noted by 66 out of 96 sCJD (68.8%) patients, followed by cerebrallum symptom (9.3%), psychiatric symptom (8.3%), cortical blindness (5.2%), pyramidal and extrapyramidal symptoms (4.2%) and other symptoms (4.2%). The median survival times of different groups varied from 3.1 to 7.1 months, but without statistical difference (*P* = 0.571). Subsequently, we analyzed the relationship of the survival time with the frequencies of the four main manifestations (myoclonus, visual or cerebella disturbance, pyramidal or extrapyramidal disfunction and akinetic mutism), which were included in the diagnostic criteria for sCJD. Although the median survival times of the patients with four signs were slightly shorter than those with fewer signs, there was no significance among the tested groups (*P* = 0.876).

**Table 4:** Clinical Characteristics and the Median Survival Times (Months) in Fatal sCJD Cases.

	Number (*%*)	Median survival time (*range*)
Foremost symptoms		
Progressive dementia	66 (68.8)	7.1 (1.0–22.4)
Cortical blindness	5 (5.2)	5.0 (2.0–16.2)
Psychiatric symptom	8 (8.3)	6.1 (1.0–20.3)
Cerebellum syndrome	9 (9.3)	5.0 (3.1–23.3)
Pyramidal and extrapyramidal symptoms	4 (4.2)	6.1 (5.0–13.2)
Others	4 (4.2)	3.1 (3.0–13.2)
* P*	–	0.571
The frequences of main clinical manifestation		
having four clinical features	10 (10.4)	5.1 (1.0–20.3)
having three clinical features	34 (35.4)	7.1 (1.0–21.3)
having two clinical features	52 (54.2)	6.1 (1.0–23.3)
* P*	–	0.876

### Relevance to the Interval Times from the Onset to Lumber Puncture in the Subtype of sCJD

CJD patients may appear different onset and symptoms progression, which may affect the interval time from the appearance of the foremost symptom to the time of seeking medical aid. To see the influence of this interval time on the survival times of the patients with sCJD, we collected the information of the times of the first lumbar puncture of 96 fatal sCJD cases and took those data as the times of medical aid. Based on the interval times, 96 sCJD cases were divided into three groups, <2 months, 2–3 months and >3 months. The median survival time of the group of <2 months (4.1 months) was much shorter than that of the groups of 2–3 months (6.1 months) and of >3 months (10.1 months), showing significantly statistical difference in the median survival time among those three groups (Table 5, *P* = 0.001). It may highlight that the patients with acute onset and rapid symptom progression may have short survival times. Furthermore, we collected the individual duration times of 96 sCJD patients after performances of lumber puncture. Although the median rest duration time (from lumber puncture to death) of the patients with short interval (<2 months) at onset was shorter than those of other two groups, there was no statistical difference among three groups (*P* = 0.646). Further analyses of clinical data found that almost all patients in those three groups appeared multiple and severe clinical manifestations at the time of lumber punctures.

**Table 5:** Interval Time (Months) from Onset to Lumber Puncture and the Median Survival/Rest Duration Times (Months) in Fatal sCJD Cases.

Interval time (*from onset* *to lumber puncture*)	Number (*%*)	Median survival time fromonset to death (*range*)	Median rest duration time fromlumber puncture to death (*range*)
<2	21 (25.6)	4.1 (2.0–17.3)	2.4 (0.3–15.4)
2–3	30 (36.6)	6.1 (3.0–23.3)	3.4 (0.1–20.5)
>3	31 (37.8)	10.1 (4.1–22.4)	3.7 (0.1–13.6)
*P*	–	0.001	0.646

## Discussion

In the present study, we have investigated the survival times of the 121 patients with various subtypes of prion diseases and evaluated the relationship of the survival time with the relevant events. The median survival time is 7.1 months (range: 1.0–23.3) and 78.5% cases died within one year after onset. Similarly, a study of 123 patients with prion disease in Sweden shows that 74.6% of patients died within one year [7]. As the predominant subtype of prion disease, the median survival time of sCJD cases in this study is 6.1 months (range: 1–81) and 74.0% of the patients survive less than one year. These data are comparable with that of a previously study from EUROCJD (European Creutzfeldt-Jakob Disease Surveillance Network) involving 2451 sCJD patients died from 31 December 1992 to 31 December 2002, in which the median survival time was 5 months (range: 1–81) and 85.8% patients died within one year [8]. However, a recent survey from Japanese CJD surveillance program has revealed a longer duration of Japanese patients with prion diseases, in which the mean disease duration of 855 patients is as long as 17.4 months and only 46.0% of them died within 1 year [9].

The survival times of 27 cases of various genetic prion diseases in this study vary distinctly. Compared with sCJD patients, 13 fCJD cases present obviously short survival times, while 12 FFI cases seem to survive much longer. Human fCJD consists of more than twenty different point-mutations and insertions in *PRNP*, which may display different clinical manifestations and disease durations. The survival times of fCJD may range from a few months to several years [10], [11]. Among 13 enrolled fCJD cases in this study, 8 are T188 K fCJD cases with median survival time of 3.0 months (range: 2–9). Such short survival times of T188 K fCJD cases may influence the entire survival times of Chinese fCJD patients, resulting in obviously shorter survival time compared with other reports [12], [13]. The survival times of Chinese FFI patients are long related to those of fCJD and sCJD ones, showing similar appearance as other studies [14]. The difference in survival times among various subtypes of human prion diseases may indicate the great impact of different prions on the disease duration. Meanwhile, all FFI cases are younger than 70 years old at onset, which might also contribute to its relatively long survival time.

Our previous surveillance data has illustrated a slightly more male sCJD cases in China (the male-to-female ratio is 1.27∶1) [4]. No statistical difference in the disease duration is observed between two genders, though the median survival time of male sCJD patients is longer than that of female. Although Japanese female sCJD cases seem to have a relatively long mean disease durations than males [9], there is also no statistical difference. Some studies have revealed that age at onset partly influence the survival time of patients with prion disease, implying that earlier disease onset is associated with longer disease duration [8], [9]. This trend seem also to be associated with the observation of the median survival times in the context of the whole prion diseases of current study, though no significance in survival times has been addressed among various age groups. Unlike the data of Japan [9], the sCJD patients in the group of 50–70 years old have relatively long median survival time. The reason is unknown. Except for limited cases younger than 50 years old, relatively rapid onset (from the symptoms appearance to first doctor-visiting) in this group might be one of the explanations.

PSWC on EEG, positive in CSF 14-3-3 [6] and typical change in MRI [15] are important criteria for the diagnosis of prion diseases. Our results here illustrate that none of the abnormal findings in those examinations shows significant impact on the survival times, although the median survival time seem to be slightly short in the context of the whole prion diseases. PSWC on EEG is usually believed to be detectable in the middle and late stage of sCJD [16]. If sCJD patients received EEG examination at onset and underwent regular review, PSWC on EEG was able to reflect disease duration to some extent. Unfortunately, most of the sCJD cases here have received only one time EEG examination during their clinical courses, which may affect the actual meaning of PSWC on EEG for disease duration. MRI imaging signal alterations might contribute to the earlier identification of the whole spectrum of sCJD cases [15]. In the group of sCJD, the patients with typical MRI images seem to have shorter median survival time, but without significance compared with those with atypical MRI images. However, four fCJD cases with typical MRI images show significantly shorter survival times than five fCJD cases with atypical MRI images. Similarly, positive of 14-3-3 protein in CSF fails to affect the durations of sCJD cases, but somehow influence the survival times of fCJD ones. The exact significances of typical MRI images and positive of CSF 14-3-3 on the disease durations of human prion diseases, especially of fCJD cases, needs more broad surveys.

The foremost symptoms and clinical manifestations of sCJD may vary largely [17]. In line with the observations in Japanese sCJD cases [9], our data here also does not figure out any statistical difference in survival time of Chinese sCJD patients among various foremost symptoms. Besides dementia, other four clinical symptoms are included in the diagnostic criteria for sCJD, including myoclonus, visual or cerebellar disturbance, pyramidal or extrapyramidal dysfunction and akinetic mutism [4]. Our previous study has identified that more percentages of sCJD patients having four or three symptoms during clinical courses show both abnormalities in CSF 14-3-3 and PSWC on EEG, possibly linked with more damage in brains. However, there is no statistical difference in the survival times among the Chinese sCJD patients having four, three and two major symptoms, although the median survival time of the patients having four symptoms is relatively short. We have to admit that the clinical manifestations are mainly collected during the hospitalizations. Large portion of the patients died in local small clinics or at home, in which the exact clinical information is hard to gather. More careful follow-up will help to understand the linkage between clinical symptoms and disease duration. Interestingly, our data here illustrate that the sCJD patients with acute onset and rapid progression have significantly shorter survival times. It may reflect more rapid brain damages in the early stage will dramatically shorten the survival times. Meanwhile, we also figure out that the rest duration times after operations of lumber puncture are comparable among the sCJD patients with distinct interval times at onset period (from onset to lumber puncture). In fact, at that time (lumber puncture), almost all patients have displayed obvious dementia and severe neurologic symptoms, which might highlight that the duration times is relatively fixed when more comprehensive brain damage occurs.

Good medical care is believed to be helpful for prolonging the CJD patients’ lives. To assess this potential impact on the survival time, we simply classify the enrolled cases based on their residences and continue medical care. No significance in survival times has been addressed between those groups, even the patients living in countryside and died at home showing relative long median survival times. Certainly, it cannot be simply excluded the possible influence of subsequent suitable medical care on the survival times of CJD patients with the classifications in this study. It may still somehow reflect the incurable features of prion diseases.

Besides our analyzed influencing factors, some other factors mentioned by previous reports may also affect the survival times of CJD patients; even imply the origin of sCJD. Neurosurgery without dura mater graft is the primary underlying risk factor for CJD infection, including iCJD and sCJD [18], [19], [20], [21], [22], [23], [24], [25]. However, none of the enrolled cases in present study has neurosurgery history that makes the evaluation for this potential risk factor impossible. One early retrospective survey published in a Chinese journal has proposed that in 38 pathologically confirmed Chinese CJD patients, two of them have had brain surgery histories one year earlier before the onset, who exhibit rapidly clinical progression and relative short survival time (approximately 2.5 months and 5 months) [26]. Linkage between positive family history of dementia (PFHD) and sCJD has been studied in a couple of reports [22], [27]. In one recently relevant studies, PFHD has been found in 12.1% of sCJD patients and 5.6% of controls (*P*<0.001), meanwhile, compare to controls, ApoE4 allele frequency (*P* = 0.005) and proportion of ApoE4 allele carriers (*P* = 0.019) are significantly higher in sCJD with PFHD [27]. Howbeit, PFDH has not recalled in all sCJD patients in this study. The codon 129 polymorphism of *PRNP* is a well-known factor to influence on the susceptibility and clinical progression of Western sCJD patients. It is also found that *PRNP* 129 polymorphism is related with AD in a polish study [28], [29], but not related with AD and amyotrophic lateral sclerosis (ALS) in other study [29]. Absolutely predominant M/M homozygote at codon 129 among either Chinese normal population or sCJD patients [4] may imply a less effective agent for occurrence of sCJD in Chinese, especially in Han Chinese. Recently, the association between *PRNP* 1368 polymorphism, which locates at about 24 kb upstream of the coding region *PRNP*, and occurrences of human sCJD and other neurodegenerative disorders have been evaluated. Dutch researchers have found a significant association of 1368T allele (*P* = 0.041) and T/T (*P* = 0.018) genotypes with Dutch sCJD, and TC-AG genotype of *PRNP* at position1368 and codon129 act as strong protective factor [30]. UK and German studies have proposed that *PRNP* 1368 is association with local sCJD cases, but was independent of the genotype of *PRNP* M129V [31], [32]. However, a Korean study has concluded no differences in genotype and allele frequencies of *PRNP* 1368 between sCJD patients and controls [33]. Although the polymorphism in the regulatory region of the *PRNP* gene may play some role in the susceptibility to sCJD, it shows obvious ethnic-related diversity. Further screening of *PRNP* 1368 polymorphism and other neurodegenerative diseases associated genes, such as ApoE4 allele frequency, in Chinese sCJD patients may help to understand their potential contributions to the occurrence of sCJD.

Although the current study is by far the largest to date to assess the survival time of prion diseases in China, there are clear limitations. One is that only a few definite sCJD cases enrolled in our study due to the low autopsy rate, leading to PrP^Sc^ molecular typing unavailable. Very limited brain biopsy assays reveal at least two molecular sub-types, including MM1 and MM2 in Chinese sCJD cases. MM2 sCJD patients in Western countries tend to have a relative long survival time [34]. The ratio of MM2 cases and the potential influence of MM2 subtype on the median survival time of Chinese sCJD patients remain unclear. In addition, 9 cases are excluded who are initially diagnosed as probable sCJD, but the revised diagnosis is based on the appearances of other diagnoses during follow-up. Hence, in the absence of biopsy or autopsy the possibility of misdiagnosis is hard to rule out, that may also influence the accuracy of the survival time of sCJD patients.

## Supporting Information

Table S1 Nine Excluded Cases with Other Diagnoses Which Was Initially Diagnosed as Probable sCJD.
(DOC)
