# Supplementary material for: Analyses of the Survival Time and the Influencing Factors of Chinese Patients with Prion Diseases Based on the Surveillance Data from 2008–2011
Source: PLoS One. 2013 May 6;8(5):e62553. doi: 10.1371/journal.pone.0062553 (PMC3645993; doi:10.1371/journal.pone.0062553)
Supplement: Table S1 — Nine Excluded Cases with Other Diagnoses Which Was Initially Diagnosed as Probable sCJD. (DOC) [file pone.0062553.s001.doc]

Table S1 Nine Excluded Cases with Other Diagnoses Which Was Initially Diagnosed as Probable sCJD

|  | Case number | Final diagnosis by follow-up | Initial diagnosis |
| --- | --- | --- | --- |
| Deceased | 1 | Cerebral infarction | sCJD |
|  | 2 | Cerebral hemorrhage | sCJD |
| Still alive | 3 | Tubercular meningitis | sCJD |
|  | 4 | Nerve system infection | sCJD |
|  | 5 | Encephalatrophy | sCJD |
|  | 6 | Encephalatrophy | sCJD |
|  | 7 | Cerebral infarction | sCJD |
|  | 8 | Cerebral infarction | sCJD |
|  | 9 | Encephalatrophy | sCJD |
